# Efficacy of Sodium Bicarbonate-Buffered Local Anesthetic Solution in Cases Requiring Bilateral Maxillary Premolar Orthodontic Extraction: A Comparative Split-Mouth Study

**DOI:** 10.7759/cureus.37934

**Published:** 2023-04-21

**Authors:** Mohamed Umer E Valiulla, Rajshekhar Halli, Saurabh Khandelwal, Ananya Mittal, Akanksha Singh, Kajal Bhindora

**Affiliations:** 1 Oral and Maxillofacial Surgery, Bharati Vidyapeeth Dental College and Hospital, Pune, IND

**Keywords:** rapid onset, alkalization, stinging sensation, pain on injection, local anesthesia

## Abstract

Aims and objectives

This study was carried out to evaluate the efficacy of 8.4% sodium bicarbonate-buffered local anesthetic solution and conventional local anesthetic in patients requiring bilateral maxillary orthodontic extractions in terms of pain on injection, onset of action, and duration of action.

Methods

102 patients requiring bilateral maxillary orthodontic extractions were included in the study. Buffered local anesthetic was administered on one side while conventional local anesthesia (LA) was administered on the other side. Pain on injection was measured using a visual analogue scale, while onset of action was measured by probing the buccal mucosa after 30 seconds of administration and duration of action was measured by the time after which the patient experienced pain or took a rescue analgesic. The data was statistically analyzed to determine the significance.

Results

The pain during injection was found to be lesser at sites where buffered local anesthetic was administered (mean visual analogue scale (VAS) score = 2.4) as compared to conventional local anesthetic (mean VAS score = 3.9). The onset of action was faster with buffered local anesthetic (mean value = 62.3 seconds) as compared to conventional local anesthetic (mean value = 157.16 seconds). Lastly, the duration of action was found to be longer for buffered local anesthetic group (mean value = 225.65 minutes) as compared to conventional local anesthetic (mean value = 187 minutes).

Conclusion

8.4% sodium bicarbonate-buffered local anesthetic was found to be more efficient than conventional local anesthetic in terms of reduction in pain on injection as well as faster onset and longer duration of action.

## Introduction

Local anesthesia (LA), over time, has made performing surgical procedures easier for healthcare professionals and leads to a better treatment experience for the patients. It prevents pain during surgical procedures by numbing a specific part of the body. Its effects are short lived; hence, healthcare professionals often use it for minor outpatient procedures. The first procedure in LA was done by Karl Kohler when he used cocaine [1]. Nowadays, amongst anesthetic agents, lidocaine is widely used. The average duration of action on soft tissue ranges from 170 to 190 minutes [2]. However, it is also a known fact that some patient,s experience burning or stinging sensation during administration and also pain during injection.

Lignocaine 2% with epinephrine 1:100,000, is the most common amide anesthetic used when giving local-infiltration anesthesia. It has a rapid onset and a moderate duration of action. Its low dissociation constant (pKa) and high lipid solubility are both factors that influence the quick onset of action [3]. Yet another one of the recent concepts introduced in LA was the use of sodium bicarbonate for alkalization of local anesthetic agent. This concept was introduced to neutralize the burning sensation and reduce pain on injection [4].

Lignocaine is the most common choice for most healthcare professionals. It is an effective anesthetic, with a rapid onset and an excellent safety record. It is very effective and free of any untoward side effects, although, administration of lignocaine can be painful [5].

A plethora of factors influence the pain of injection, including the introduction of needle, rate of injection, pressure from the fluid distention of the tissue, and the pH of the solution [6,7]. Lignocaine is unstable at its pH of 7.9. Therefore, it is formulated in an acidic pH to increase its stability and shelf life. The resultant pH is 4.7. This is well below the physiological pH and can lead to irritation of the tissue that may be perceived by the patient as a stinging or burning sensation [7,8].

Sodium bicarbonate can be added to lignocaine in order to increase the pH of the anesthetic solution to reduce the burning or stinging sensation [9]. Several studies have been performed on addition of sodium bicarbonate to lidocaine, suggesting that it leads to reduction in pain during injection and aids in faster onset of action [10]. This study was conducted to evaluate the efficacy of 8.4% sodium bicarbonate buffered local anesthetic against conventional 2% lignocaine with 1:80,000 adrenaline with respect to its pain on injection, onset of anesthesia, and duration of action.

## Materials and methods

102 patients requiring bilateral extractions of maxillary premolar teeth for orthodontic treatment were selected. The study was conducted by a split mouth method. The extractions were performed over a single appointment, wherein non buffered 2% lignocaine with 1:80,000 adrenaline was administered to one side (control group), while 2% lignocaine with 1: 80,000 adrenaline freshly buffered with 8.4% sodium bicarbonate in 10:1 ratio was administered on the other (study group). The study was approved by Bharati Vidyapeeth (Deemed to be University), Dental College and Hospital, Pune, India Institutional Research Committee with IRB number BVDU/DCH/680-1/2020-21. Both the operator and the patient were blinded to the solutions used. Patients included in the study were aged 14 years or older of either gender, who had bilateral maxillary premolar teeth indicated for orthodontic extraction, who were willing to participate, and who were free of any significant systemic diseases. Patients excluded from the study consisted of medically compromised patients, patients having known allergy to lidocaine, mentally challenged patients, patients unable to communicate, pregnant and lactating women, and patients unwilling to be a part of the study or unable to come for follow up.

Informed consent was obtained from all the patients. The operator was blinded to the solutions that were to be administered to avoid bias. One syringe contained conventional local anesthetic solution while the other syringe contained buffered local anesthetic solution. The first parameter to be measured was pain on injection, which was measured on a visual analogue scale (VAS). It is a 10-point scale, where a score of 0 equals no pain and 10 equals very severe pain. The VAS score was entered after the procedure by showing the VAS scale to the patient. Further, onset of action was measured as the time taken for the anesthetic solution to act. It was measured by probing the buccal mucosa after 30 seconds of administration, in regular intervals of five seconds till the patient did not feel the probe. Lastly, the duration of action was recorded on the subsequent visit by asking the patient the time after which he/she experienced pain or the time when he/she consumed a rescue analgesic.

All the data obtained was subjected to statistical analysis (Mann-Whitney U Test).

## Results

**Figure 1:**
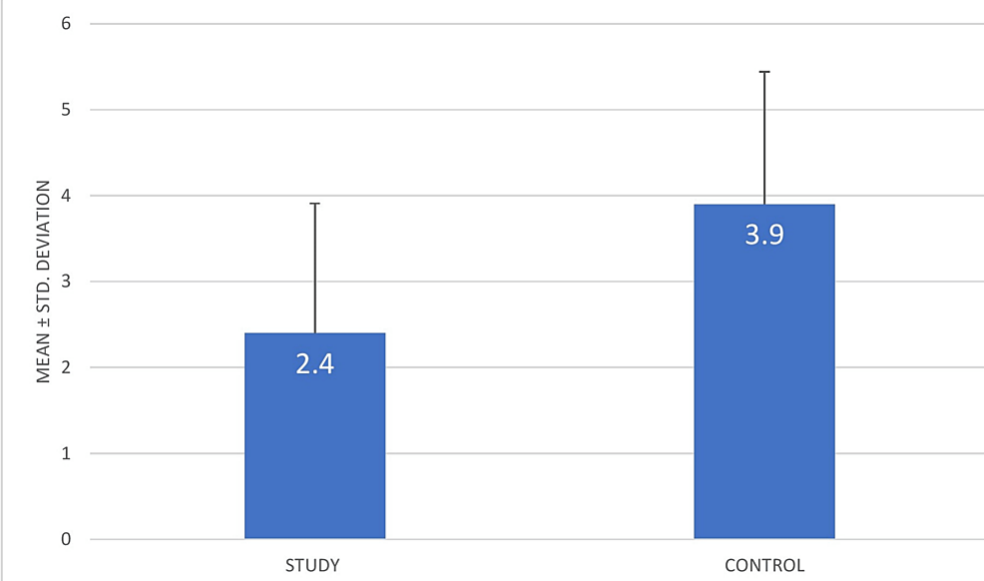
Mean and SD of pain on injection (mean VAS score) VAS:

The total sample size on which the parameters were assessed was 102 (204 sites). The pain during injection was found to be lesser at sites where buffered local anesthetic was given in 72.56 % of patients, whereas 18.62 % of patients found buffered anesthesia to be as painful as non buffered anesthesia. However, 8.82 % of patients found buffered anesthesia to be more painful than non buffered. The descriptive statistics for pain in terms of mean VAS score has been provided in Table 1. The minimum pain level for the study group was 0 while for the control group, it was 1. The maximum pain level for the study group was 6 while it went up to 8 in the control group. The mean and SD were 2.40 and 1.51, respectively, for the study group and 3.90 and 1.54, respectively, for the control group (Figure 1).

**Table 1:** Descriptive statistics of pain (mean VAS score) VAS:

Group	Minimum	Maximum	Mean	SD
Study	0.00	6.00	2.40	1.51
Control	1.00	8.00	3.90	1.54

**Figure 2:**
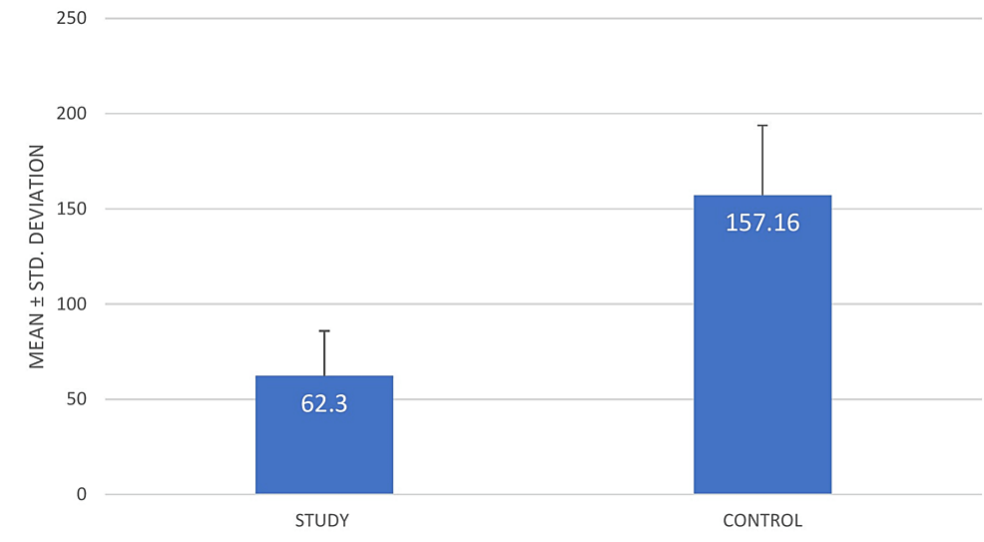
Mean and SD of onset of anesthesia (in seconds)

The onset of anesthesia of buffered anesthetic is believed to be faster than non buffered local anesthetic. Amongst the 102 patients, in 92.34 % patients, the onset of action was faster in buffered LA. On the other hand, there was no difference in onset of action between control & study in 6.68% of patients and in 0.98% of patients, buffered anesthetic took a longer time to act. Table 2 provides the descriptive statistics of onset of action of the study and control groups. The minimum onset of action of the study group was 30 seconds and the maximum was 170 seconds. For the control group, the minimum onset of action was 40 seconds and maximum was 225 seconds. The mean and SD for the study group was 62.30 and 23.60, respectively, while for the control group it was substantially higher at 157.16 and 36.69, respectively (Figure 2).

**Table 2:** Descriptive statistics of onset of anesthesia (in seconds)

Group	Minimum	Maximum	Mean	SD
Study	30	170	62.30	23.60
Control	40	225	157.16	36.69

**Figure 3:**
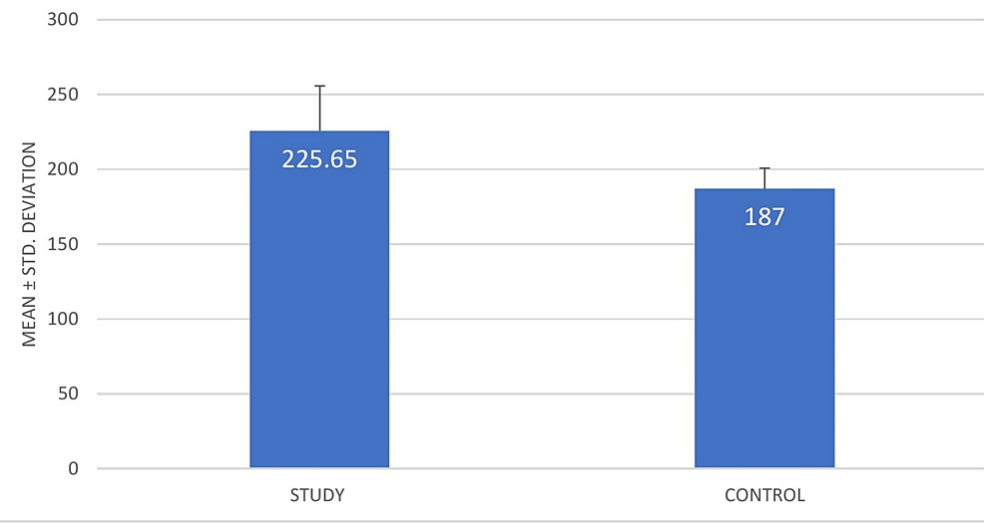
Mean and SD of duration of action (in minutes)

The duration of action was found to be more for buffered local anesthetic in 81.61% of patents, whereas the duration of action was same for both in 7.61% and lesser in 10.78% for buffered local anesthetic. Table 3 provides the descriptive statistics for the duration of action (in minutes). The minimum and maximum values for study group was 120 minutes and 274 minutes, respectively, while for the control group, it was 150 minutes and 250 minutes, respectively. In terms of mean and SD, it was 225.65 and 30.16 minutes for the study group, respectively, and 187.00 and 13.78 for the control group, respectively (Figure 3).

**Table 3:** Descriptive statistics of duration of action (in minutes)

Group	Minimum	Maximum	Mean	SD
Study	120	274	225.65	30.16
Control	150	250	187.00	13.78

The comparison between the study group and the control group with respect to the three important parameters has been conducted using the Mann-Whitney U test as the values did not follow normal distribution and it has been depicted in Table 4. The difference between them for the onset of action (in seconds) was statistically significant (p value 0.000*). A similar result was found with respect to duration of action (in minutes) (p value 0.000*) and pain score and the differences between them were found to be statistically significant as well (p value 0.000*).

**Table 4:** Comparison between study and control groups using Mann-Whitney U test *: p value < 0.05

Parameter	Mean Difference	Mann-Whitney U test	Z-value	p-value
Onset of anesthesia (in seconds)	-94.86	472.00	8.854	0.000*
duration of action (in minutes)	38.65	1473.00	11.238	0.000*
Pain (Mean VAS Score)	-1.50	2590.00	6.296	0.000*

## Discussion

Local anesthetics can be described as salts that have been dissolved in sterile water or saline. The local anesthetic medication is present in solution as positively charged (BNH+) and negatively charged (BN) molecules (cation). Only the base (BN) component of a local anesthetic solution can pass through the epineurium. Charged cation and uncharged base molecules are distributed equally when the pKa equals the pH. The quantity of solution that will cross the epineurium and be transformed into positively charged BNH+ ions, which block sodium channels, will increase in proportion to the amount of the base form present. Therefore, a low pH of a solution or tissue will result in less free BN ions and delay or diminished local anesthetic effect in cases of infections. It is particularly challenging for the normal injection to deliver appropriate anesthesia at the infection site due to lower tissue pH. Because infected tissue is more acidic, BN conversion struggles to take place [11-15]

The burning feeling that occurs during injection and the delay in action are two of the main problems that can occur after using a local anesthetic solution [11]. Commercially available local anesthetic solution's acidic content is primarily to blame for this [11,13]

To maximize stability in solution and shelf life, a commercially available local anesthetic solution must have an acidic pH [13]. An amide local anesthetic is a weak base, chemically speaking. A stable, injectable anesthetic solution is produced during production when amides react with an acid to generate a hydrochloride salt, making them water-soluble. Prior to injection, all local anesthetic solutions are acidic. The pH range without vasoconstrictors is between 5 and 7. It ranges from 3.8 to 5 when using vasoconstrictors [11,13]

Local anesthetics, however, have a number of disadvantages, such as the stinging effect they produce on injection. A degree of tissue damage upon injection is also linked to local anesthetics. Local anesthetics begin to work relatively slowly. Furthermore, in the presence of infection and inflammation, local anesthetics do not function as consistently.

Anesthetic buffering can be used to get around each of these limitations. It decreases the onset time, offers anesthetic effect of CO_2_, and eliminates injection pain, stinging, and tissue harm [14]. For nerve blocks and regional anesthesia, buffered local anesthetic solution was employed, and it was discovered that a higher pH of the solution produced superior anesthesia [16].

Alkalinization may have benefits. First, the patient might feel less stinging discomfort due to the solution's higher pH. Second, the pH of the injected solution may more quickly approximate the pH of the typical tissue following injection. The more quickly a mixture of charged and uncharged forms formed, the more quickly drug diffusion and nerve blockage begin. This may be especially helpful at body sites where there may be a delay in the pH rise after injection due to inadequate tissue buffering capacity [13].

Warming lignocaine, buffering it with a chemical agent, and warming buffered lignocaine are all alkalinization techniques [17]. An alternative to relying on the body to achieve the similar pH shift after injection is to raise the pH of local anesthetic (also known as Banesthetic buffering or Balkalinization) right before injection using a bicarbonate solution. The body will use the same chemical mechanism and molecule (NaHCO_3_) to buffer the anesthetic following the injection in vivo (within the body), but the ex vivo (outside the body) method represents a means to achieve the pH change instantly and more consistently [18].

In the world of medicine, the idea of local anesthetic buffering is widely accepted. In a systematic review by Davies on the buffering of LA, it was discovered that using sodium bicarbonate as a buffer significantly decreased injection discomfort without compromising efficacy [15]. The most popular way to buffer LA is by adding 1 ml of 8.4% sodium bicarbonate to 10 ml of local anesthetic [19]. CO_2_ and water are produced as by products of buffering with bicarbonate. Catchlove earlier verified that the interstitial fluid pH within the nerve sheath tends to decrease when CO_2_ is present [20]. As a result, the anesthetic solution's ionization in this area is further enhanced. Furthermore, because CO_2_ diffuses quickly through the nerve sheath and reaches the axon before the anesthetic solution, it has the potential to produce an immediate kind of analgesia in comparison to lignocaine. Buffered anesthetics in a glass carpule may be as useful as gas, but they may also be unstable. The freshly made buffered mixture should be delivered right away because too much alkalinity can lead to precipitation in the solution. According to Momsen et al., buffered lignocaine adrenaline solution is stable for up to 24 hours after preparation [7].

In our present study, a split-mouth method was used wherein conventional local anesthetic was administered on one side and buffered local anesthetic was administered on the other. Regarding pain on injection, most of the patients experienced less pain in sites where buffered local anesthetic was administered as compared to conventional local anesthetic. For the study group, the minimum pain threshold was 0, while for the control group, it was 1. The study group's maximal pain threshold was 6, whereas the control group's threshold was 8. The study group's mean ± SD was 2.40 ± 1.51, while the control group's was 3.90 ± 1.54. The p value was found to be 0.000* (statistically significant). In terms of onset of action, sodium bicarbonate buffered local anesthetic had a faster onset of action as compared to conventional local anesthetic. All the patients were probed in regular intervals of five seconds after 30 seconds of administration and were asked if they felt pain. The patients claimed to stop experiencing pain sooner on the sides where buffered local anesthetic was administered. However, there were a few exceptions wherein buffered local anesthetic seemed to act slower than conventional local anesthetic. The study group's mean ± SD was 62.30 ± 23.60, while they were significantly higher for the control group at 157.16 ± 36.69. The p value of the same was 0.000* (statistically significant). The dissociation constant (pKa) level of a local anesthetic plays a major role in determining when it starts to work. A lower pKa speeds up the commencement of action and improves tissue penetration. Warming and buffering may cause the pKa to change, increasing the proportion of particles in the unionized state. Unionized particles diffuse into the neuron more quickly, resulting in more speedy and efficient inhibition of nerve.

Both our results are in agreement with a study done by Kashyap et al., who evaluated the outcome of buffering 2% lignocaine with 1:80,000 epinephrine using a prospective randomized study with 100 individuals and concluded that the group receiving buffered lignocaine experienced much less injection pain (p = 0.0001) and significantly faster local anesthetic onset (p = 0.001) [21]. A randomized, double-blind research with 200 patients getting maxillary infiltrations for dental extraction was carried out by Al-Sultan et al. who concluded that patients which came with periapical lesions and had injections of buffered local anesthetic experienced much reduced discomfort upon injection, faster onset, and uneventful extraction [22]. Another randomized, double-blind research of buffered local anesthetics was carried out by Al-Sultan et al. [23]. Following statistical analysis, they discovered that the buffered solutions were substantially less painful during periapical surgery than the control solutions during injection, their onset was significantly faster, and their pain levels were significantly lower. Studies done by Agrawal et al. showed decreased time of onset of anesthetic action [24]. There was no significant difference in duration of action of anesthesia and return of pain sensation. Lingaraj and Vijaykumar have also shown no difference in onset and duration of action; however, they found that buffered solution has a faster onset of action in infected cases [25].

In a study done by Siler et al., there were no appreciable differences between buffered and non-buffered solutions in terms of the spread of analgesia, the length of the process, the duration of total anesthesia, and the regression of anesthesia [26]. They looked at 2% lignocaine administered as 17-23 mL epidural injections with or without sodium bicarbonate in a 10:1 ratio. Additionally, Gaggero et al. investigated the effects of 2% lidocaine diluted 10:1 with saline, 2% lidocaine diluted 10:1 with 8.4% sodium bicarbonate for injection right away, and 2% lignocaine diluted 10:1 with 8.4% sodium bicarbonate for injection an hour beforehand and discovered no appreciable variations in the efficacy of epidural anesthesia [27]. Furthermore, according to Sinnott et al., adding sodium bicarbonate to 1% lidocaine reduced the depth and length of anesthesia by 25% and 50%, respectively [28]. They discovered that adding sodium bicarbonate to epinephrine-containing fluids hastened the onset of anesthesia while leaving the degree and duration intact.

The duration of action too was longer on sides where buffered LA was administered. It was measured on the subsequent visit by asking the patient the time when he/she started experiencing pain or consumed a rescue analgesic. The study group's minimum and maximum values were 120 minutes and 274 minutes, respectively, while the control group's values were 150 minutes and 250 minutes, respectively. The study group's mean ± SD were 225.65 ± 30.16 minutes and 187.00 ± 13.78 minutes, respectively, while p value was found to be 0.000* (statistically significant). This result is in agreement with a study done by Afolabi et al., who investigated the effect of buffering lignocaine on pain on injection and duration of action and concluded that buffering lignocaine increased the duration of its anesthetic effect [29]. 

Although our sample size is relatively larger than the previous studies performed, we would recommend further studies on a much larger sample size for a more comprehensive outcome. Furthermore, our study was performed in cases requiring bilateral orthodontic extractions with no infection. However, routine extractions also consist of teeth with periapical infections and hence, more studies should be conducted with respect to infective cases to determine the efficacy of buffered local anesthetic solution in the same.

## Conclusions

Advancement in LA is an evergreen prospect in the field of maxillofacial surgery and dentistry as a whole. The ultimate goal is to provide to the patient a painless and pleasant treatment experience and this goal starts with administration of painless anesthesia. Out of all the available modalities, buffered LA seems to be a viable, and at the same time, an economical alteration that could be made to conventional LA for making the administration more pleasant for the patient. Our study proves that buffered local anesthetic is significantly better than conventional local anesthetic owing to its lesser pain on injection, faster onset of action and longer duration of action. The application of buffered LA in dentistry should therefore be looked into as a possible solution in reducing patient discomfort during injection and achieving a faster anesthetic effect. Further studies could also be conducted in the future to explore the utility of buffered LA in infective cases because of its raised pH.
